# Ontogenetic shift from aposematism and gregariousness to crypsis in a Romaleid grasshopper

**DOI:** 10.1371/journal.pone.0237594

**Published:** 2020-08-20

**Authors:** Emma Despland

**Affiliations:** Biology Department, Concordia University, Montreal, Quebec, Canada; USDA Agricultural Research Service, UNITED STATES

## Abstract

Traits of chemically-defended animals can change as an individual grows and matures, and both theoretical and empirical evidence favour a direction of change from crypsis to aposematism. This study examines the suite of traits involved in an unusual opposite shift from aposematism to crypsis in a neotropical toxic-plant-feeding Romaleid grasshopper, *Chromacris psittacus* (Gerstaecker, 1873). Field surveys, behavioural observations and a rearing experiment compare host plant choice, aggregation, locomotion and thermoregulation between life history stages. Results showed that both nymphs and adults fed exclusively on a narrow range of Solanaceae plants, suggesting that the shift in defensive syndrome is not due to a change in chemical defense. Instead, nymphal aposematism appears linked to aggregation in response to plant-based selection pressures. Slow nymphal development suggests a cost to feeding on toxic plant compounds, and grouping could mitigate this cost. Grouping also increases conspicuousness, and hence can favour warning colourating in chemically-defended insects. The role of diet breadth in aposematism is poorly understood, and these results suggest how constraints imposed by feeding on toxic plants can generate bottom-up selection pressures shaping the adaptive suites of traits of chemically-defended animals.

## Introduction

Animals that feed on toxic plants are often themselves distasteful or toxic, and exhibit variable suites of associated traits, including some that enhance defense (e.g. defensive secretions, aposematism and group-living) and others made possible by lower predation pressure (e.g. large size, sluggish or conspicuous behaviour). These traits often change during ontogeny with size-dependent selection pressures [1]. Ontogenetic colour change in chemically-defended animals generally goes from crypsis early in development to aposematism at larger body size [2], and the increase in aposematism during herbivore ontogeny seems to be reinforced by both bottom-up and top-down forces [1]. Size-linked selection pressures are more likely to favour crypsis in small animals (which are both less conspicuous and less able to sequester a large enough dose of toxin to deter predators) than in larger ones [3]. An opposite shift from aposematism to crypsis could be favoured under particular circumstances where the costs of crypsis decrease over ontogeny [4]; a few examples exist (e.g. in a tropical frog [5]), notably in the Pyrgomorphidae or gaudy grasshoppers [6,7], but overall empirical studies of aposematism-to-crypsis ontogenetic shifts are rare.

**Fig 1:**
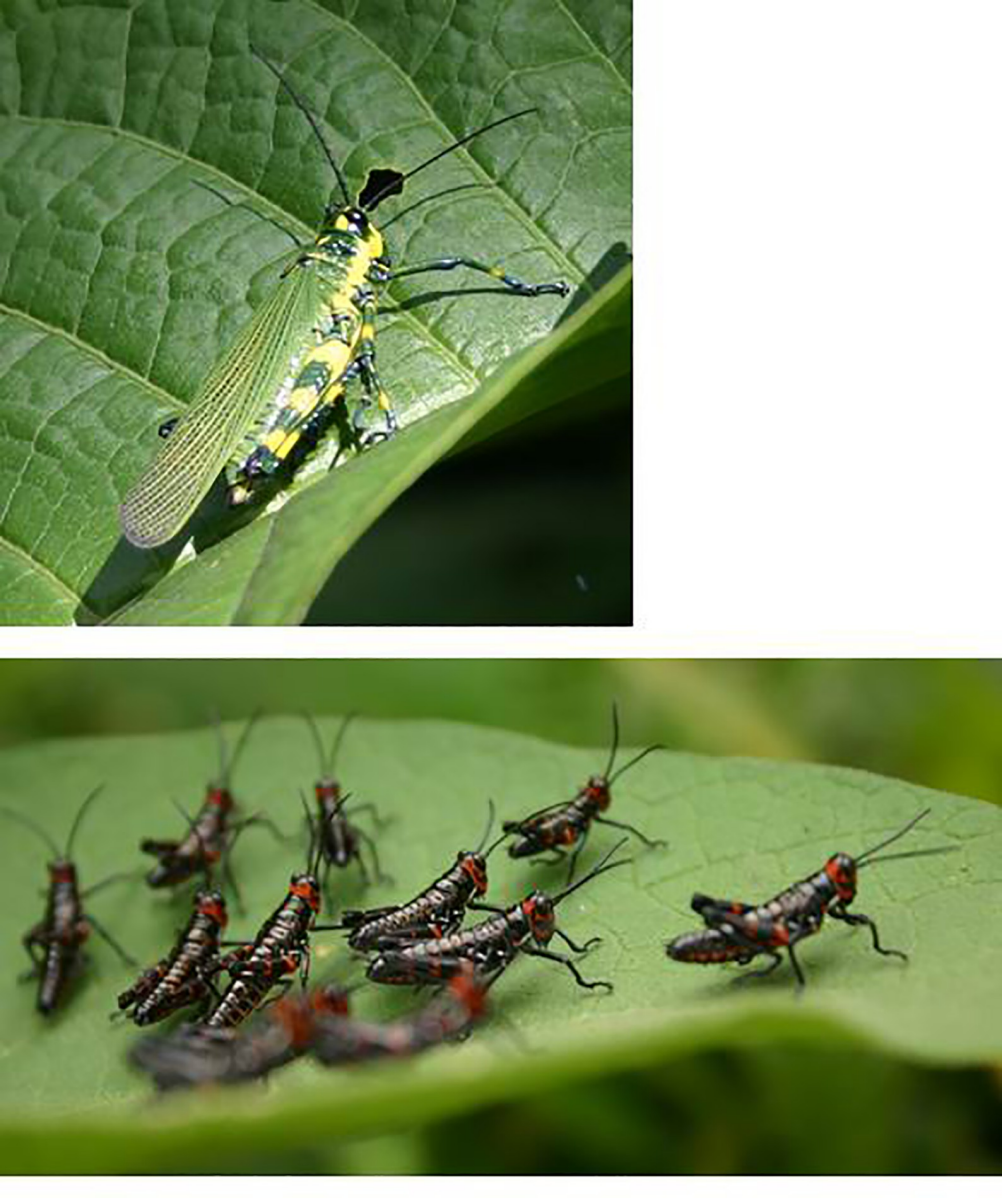
*Chromacris psittacus* adult and early-instar nymphal herd.

Such a shift from aposematic to cryptic colouration appears to occur in some Romaleid grasshoppers (Table 1; Fig 1), providing an opportunity to test how colouration interacts with other traits, and how these trait associations vary across ontogeny. Romaleid grasshoppers (ca 500 species) exhibit various degrees of chemical defense, from distastefulness to toxicity to secretion of noxious compounds, which can be integrated with other traits in diverse defensive suites [8]. Romaleids are called lubbers due to their sluggish behaviour. The two best studied lubbers, *Romalea microptera* and *Taeniopoda eques*, both sequester compounds from plants and use them in defensive secretions expelled from the spiracles in response to attack [9–11]–the quantity and quality of these secretions is highly variable and affects deterrence [9]. The Romaleidae and sister families are thought to have diverged from Old World ancestors in the Cretaceous and to have diversified in South America [12,13]; feeding on toxic plants appears to be ancestral and to have driven associated changes in physiology, morphology, life history and ecology [8]

**Table 1:** Life history traits of previously studied Romaleids. Table includes data from the literature on diet breadth, colouration and aggregation of nymphs and adults, nymphal development time, adult flight capacity and defensive secretions and final female adult size.

	NYMPHS				ADULTS						
Species	diet	color	group	develop. time	diet	color	group	flight	secrete	♀size (mm)	Source
*Rhicnoderma spp*. *(Bactrophorinae)*	P	C	s	?	p	c	s	no	?	40	[22]
*Xyleus discoideus (Procolpini)*	P	C	s	60 d. at 28°C	p	c, f	S	weak	No	50	[21]
*Tropidacris collaris (Tropidacrini)*	P	A	g	?	p	c, f	?	yes	No	100	[11,16,17]
*Brachystola magna (Romaleini)*	P	C	s	27 d. 20–25°C	p	C	S	no	No	65	[19]
*Zoniopoda tarsata (Romaleini)*	P	A	s	?	p	a	s	yes	?	60	[20]
*Taeniopoda eques (Romaleini)*	P	A	g	39 d. in field	p	a	S	no	yes	51	[11]
*Romalea microptera (Romaleini)*	P	A	g	60 d. in field	p	A	G	no	yes	76	[18]
*Chromacris colorata (Romaleini)*	O	A	g	8–10 days/ instar at 28°C	o	c,f	s	yes	No	?	[23]
*C*. *psittacus (Romaleini)*	O	A	g	2 instars in 50 days at 26:18°C	o	c, f	s	yes	No	40	present study

The subfamily (for the one species that is not in the Romaleinae) or tribe is given for each species. The genus *Taeniopoda* is thought to be paraphyletic with respect to *Romalea microptera* [24]. Recorded nymphal development times are given (d. = days), with temperature conditions under which these measurements were made. Question marks indicate fields for which no information was found. Codes: diet: p–polyphagous, o–oligophagous; colour: a–aposematic, c–cryptic, f–flash; grouping: g–gregarious, s–solitary (‘solitary’ indicates that no evidence exists for active aggregation, although the species may occasionally reach very high densities and be important crop pests). Secrete = secretion from specialized glands only, not regurgitation.

Many lubber species exhibit warning colouration at some stage in the life cycle and some are also gregarious. Lubbers are generally sluggish, some are flightless, and exhibit exposed diurnal behaviour, combined with behavioural thermoregulation and basking. They attain very large sizes, presumably linked with an absence of vertebrate predation [8]. The few lubber species studied to-date combine these traits in a variety of suites of traits [8]–see Table 1. Some variation in traits appears easily explained by local adaptation: for instance, desert-dwelling *T*. *eques* exhibits behavioural thermoregulation and early morning basking [14], but subtropical wetland *R*. *microptera* does not [15].

Distasteful lubbers obtain their defensive compounds from host plants [25], and, in the one species studied (*T*. *eques*), nymphs and adults show the same pattern of feeding and diet breadth [26]. However, other selection pressures vary during ontogeny: as insects grow bigger, thermal mass increases and basking need decreases–as expected, *T*. *eques* adults show less thermoregulatory behaviour than nymphs [17]. Predation risk has also been shown to decrease with increasing size in warningly-colored distasteful lubbers, as larger chemically-defended prey are more avoided by predators [8,27]. Some predators attack small nymphs (that contain a smaller dose of toxin) but not adults [17]. What limited evidence exists on romaleid ontogeny suggests that, among species with aposematic nymphs, some retain warning colouration as adults (e.g. *T*. *eques*, *R*. *microptera*) whereas others (e.g. *Chromacris* species) exhibit an unusual ontogenetic shift, switching to crypsis with flash-colouration as adults: the body is cryptic, but in flight the colourful hindwings appear [23,28–33]–see Fig 1. Adults appears to combine low contrast with background vegetation and disruptive patterns, such that they are difficult for humans to detect on vegetation [29].

Several possible scenarios could underlie this unusual ontogenetic colour change: the simplest involves an ontogenetic change in host plant use that impacts effectiveness of chemical defense (1)–indeed, several Orthopteran species are aposematic as nymphs feeding on toxic plants but become cryptic as adults when they change their diet [3]. Alternatively, chemical defense could remain throughout the life-cycle, and aposematism could be favoured early in ontogeny by aggregation driven by other selection pressures, e.g. thermoregulation, or overcoming plant defenses [34]. These pressures are generally stronger in smaller animals [1].

The following questions were investigated in order to compare suites of traits of *Chromacris psittacus* adults and nymphs and to assess the above scenarios regarding ontogenic trajectories:

1. Host plant choice: do nymphs and adults feed on the same range of plants and/or plant parts? Do they exhibit diet mixing [35] to enhance their chemical defense?
2. Aggregation: Do nymphs actively stay together (truly gregarious sensu Costa[30]) or are groups simply resource-driven aggregations [36]? Specifically, we will test whether synchronization keeps nymphs together during movement [37,38].
3. Underlying benefits of nymphal aggregation: do nymphal groups exhibit behaviours that suggest either thermoregulatory or plant-based advantages to grouping? Specifically, do they bask collectively to increase temperature and maximize growth rate [14]? Do they synchronize feeding to overcome plant defenses [39–41]?
4. Sluggishness: Do adults/nymphs exhibit sheltering or exposed behaviour? How mobile are they? Is movement sluggish?
5. Nymphal growth rate: do nymphs grow fast to attain large size and escape invertebrate predators as per the slow-growth-high-mortality hypothesis [8,42]?

## Methods

The hypotheses listed above were tested with a combination of a field survey, field observations and laboratory rearings. Data were combined from these three approaches to characterize the *C*. *psittacus* ontogenetic strategy.

### Study species

The genus *Chromacris* (9 species, several subspecies) feed on Solanaceae host plants, and are common in disturbed areas in the neotropics [43]. The black-and-red colouration of *C*. *psittacus* nymphs is typical of aposematism as it increases conspicuousness against the green of foliage [44], whereas the green-and-yellow of adults blends in with the environment [29]–see Fig 1. Adults also display flash colouration: the bright orange hind wings appear when they fly away in response to disturbance [33]. Nymphs undergo 6 instars before the moult to the adult stage [23]. *C*. *psittacus* adults shows no evidence of specialized defensive secretions but do regurgitate when handled (personal observation). The species is thought to be distasteful to predators [23,28,29,45]; distastefulness is more likely derived from gut-contents than from sequestered compounds [46,47], although this has not been tested.

### Field survey of nymphs and adults

A field survey of adult and nymphal *C*. *psittacus* was conducted in 2017 in semi-natural partially shaded areas (overgrown old-fields, forest edges) in the Mindo valley (00∘03′44.1′′S 78∘45′41.7′′W), located in cloud forest at 1250 m a.s.l. on the Western slope of the Andes in the province of Pichincha, Ecuador.

The following variables were recorded for each individual or group observed: host plant, leaf position (expanding, mature or senescent), group size and whether the insect was in the sun or shade. Nymphs were scored for developmental stage: early instar = no visible wing pads, mid-instar = two pairs of wing pads visible or late instar = only forewing pads visible and beginning background colour change to green instead of red. Each host plant on which an individual or group was observed was flagged, and monitored daily to record changes in group size or movement between host plants. Field work was conducted on private land, authorized by land-owner (Maria Elena Garzon Jaramillo).

These data were used to test hypotheses about: 1. Host plant choice (are nymphs and adults observed on the same host plants?), 2. Aggregation (what is the frequency distribution of nymphal group sizes?), and 3. Sluggishness (do individuals react to the presence of the observer?).

### Behavioural observations

The behaviour of 12 replicates (nymphal groups or adult individuals) was recorded in the field using Noldus Pocket Observer. Three classes of behaviour were continuously recorded, each comprising several mutually exclusive states: activity (resting, moving or eating), location (on host plant, on another plant or off plants) and basking. The basking variable was scored as follows: in sun (the weather is sunny and the insect is in direct sunlight), in shade (the weather is sunny and the insect is in the shade), overcast or light rain (in these two cases the question of basking behaviour does not arise). Observations were discontinued under heavy rain. Several point behaviours (events with no duration) were also recorded: flight (adults only), changing leaf and changing plant.

Observations were intended for 2 h each, but some were terminated early due to interruptions (e.g. by rain or the insect flying away and getting lost). Others were continued for longer, for a total of 24h of observation.

The behaviour of nymphal groups was recorded in a similar fashion as that of adult individuals, except that records also included the number of individuals involved in the behaviour. These data were used to test hypotheses about:

1. Host plant choice: diet-mixing was tested by recording each plant on which the individual/group fed during the course of the assay [35].
2. Aggregation: Active aggregation of nymphs was evaluated as synchronization of movement by testing for overdispersion relative to a binomial process [48]. For each replicate observation, the frequency distribution of the number of nymphs engaged in moving was compared with a binomial distribution around the mean number of moving individuals over the course of that observation. The goodness of fit to the binomial was tested with an overdispersion index, calculated as the ratio of deviance to the degrees of freedom. If behaviour of nymphs in the herd is independent (i.e. no synchronization), this index is equal to 1 [49]. A chi-square test was used to assess significance of the departure from independent behaviour [47].
3. Thermoregulation: periods of each observation in which weather was overcast or light rain were excluded from the analysis. Proportion of observation time spent in the sun was compared with that in the shade to test whether insects bask to increase their body temperature. Quasibinomial distribution was used because the data did not fit the binomial.
4. Overcoming plant defenses: As in 2, chi-square was used to test if feeding is synchronized based on goodness of fit to a binomial.
5. Sluggishness: Proportions of time spent moving and feeding were compared between nymphs and adults with quasibinomial GLMs.

### Nymphal rearing

Ten early-instar nymphal herds (mean group size 9.6 +/- 2.2 S.D.) were collected in the field during October and November 2017 and reared in sleeve cages on *Brugmansia* spp (mean rearing period 50 days +/- 10 S.D.). Each herd was weighed with a portable balance (Ohaus Scout SPX123). Due to the low resolution of the balance (10 mg) and the gregarious behaviour of the insects, the group was weighed as a whole, and mass was divided by the number of individuals to calculate an individual relative growth rate, RGR (mg.day^-1^.g^-1^). Rearing was done under field conditions with a 12:12 photoperiod, 18°C: 26°C night- and day-time temperatures.

## Results

### Host plant use and diet mixing

In the field survey, of 60 adults seen, the sex ratio was balanced (32 females, 28 males). Most were observed on *Solanum* section *geminata* spp (N = 21) or *Brugmansia* spp (N = 20), but a few were on other Solanaceae as well (*S*. *acerifolium* (N = 5), *Cestrum* spp (N = 2), *S*. *candidum* (N = 1), *Acnistus arborescens* (N = 4), *Solanum lycopersicum* (N = 7)). All were on mature leaves. Of 30 nymphal groups seen, most were seen on *Solanum* section *geminata* spp (N = 21) and *Brugmansia* spp (N = 6). A few late instar groups were seen on *A*. *arborescens* (N = 2) and *Cestrum* spp (N = 1).

The behavioural observations showed no evidence for dietary mixing. Adults occasionally moved between individual plants, but never between species: movement between individual host plants was observed 20 times in 24h of observation. Some individuals changed host plant (N = 9 individuals moved between plants between 1 and 6 times each) but others (N = 3) never did so in the 2h observation period. Nymphal herds were never observed to move between plants. The field survey showed that nymphal herds were often observed on the same individual plant for several subsequent days, see Table 2.

**Table 2:** Field observation of nymphal herd group size (mean +/- S.E.) and persistence (number of days the group was observed on the same plant, mean +/- S.E.) per developmental stage.

instar	# groups	group size	persistence (days)
early	15	22.73 +/- 8.2	7.66 +/- 3.05
mid	7	19.14 +/- 1.80	3.43 +/- 0.64
late	8	5.13 +/- 1.38	2.00 +/- 1.10

Nymphal herds also moved from one leaf to another within the plant less often than adults (total of 16 times in 24h compared to 56 times). Adults appeared to be selective of feeding sites, moving from one leaf to another before settling to feed, whereas nymphal herds always fed on the leaf on which they were situated. Adults were observed to spend most of their time on host plants (85.6% of observation duration), and nymphs were never observed off hosts.

Both nymphs and adults showed a clear alternation between meals and intermeal intervals (sensu [50]). Adults exhibited meals of a median duration of 7 min (first quartile 2 min; third quartile 15 min; n = 27 uncensored bouts) of feeding, interspersed with 30 min (first quartile 23 min; third quartile 61 min; n = 17 uncensored bouts) intermeal intervals, time spent generally immobile with short, slow movements. Nymphal herds exhibited synchronized meals of median 23 min duration (first quartile 10 min; third quartile 32 min; n = 28 uncensored bouts), and 33 min (first quartile 18 min; third quartile 63 min; n = 18 uncensored bouts) quiescent intermeal intervals.

### Nymphal aggregation and synchronization

In the field survey, most adults were observed alone, but nymphs were always observed in groups. Group size decreased as development progressed (see Table 2).

**Fig 2:**
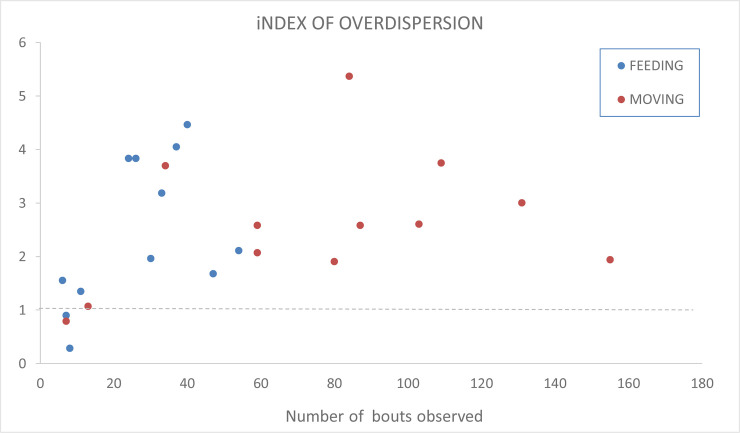
Synchronization of eating and moving bouts. The y-axis represents the index of overdispersion from the binomial distribution–values greater than one indicate synchronization of behaviour (indicated by the dashed line). The x-axis represents the number of behavioural bouts observed.

Overdispersion analysis reveals that 10 of 12 groups exhibited significant (chi-square p<0.05) synchronization of movement; the two exceptions are short observations with only approximately 10 moving bouts recorded, suggesting that the number of data points was insufficient for an adequate test of behavioural synchrony (see Fig 2). Whenever a nymph became separated from the group during movement around the plant’s architecture, it quickly rejoined the group, orienting to visual stimuli [23].

For eating behaviour, 8 out of 12 groups exhibited significant synchronization. The 4 replicates in which synchronized feeding was not observed had again been short observations in which less than 10 feeding bouts were observed (see Fig 2).

### Basking

When it was sunny, both nymphs and adults spent more time in the shade than in the sun (for both adults and nymphs P<0.0001; binomial GLM comparing proportion of assay time spent in sun or in shade)–see Table 3. When clouds moved away and grasshoppers found themselves in full sun, they generally moved to the shade within minutes (the average duration of bouts in the sun was 4.9 min for adults and 4.8 min for nymphs, compared to 22 min in the shade for nymphs and 66 min for adults).

**Table 3:** Behavioural observations, showing number of events for point behaviours (moving between leaves, between plants, and flight in response to a disturbance) and proportion of assay time spent in mutually exclusive timed behaviours (eating, moving, resting) or locations (on a host plant, on another plant, on the ground). Time spent in sun or shade is given as a proportion of the time in which the weather was sunny.

	ADULTS		NYMPHS	
Behaviour	# events	% time	# events	% time
Change leaf	56		16	
Change plant	20		0	
Fly	39			
Eating		7.33		20.8
Moving		7.41		10.8
Resting		85.3		62.2
Host plant		85.6		100
Other plant		9.70		0
Non plant		4.41		0
Shade		93.04		82.10
Sun		6.90		17.81

The persistence of nymphal colonies on the same host plant made it possible to observe their daily pattern of movement: they moved down to the ground in the evening and stayed at the base of plants overnight, where they remained until ca 10 AM the following morning, when they moved up the plant and onto a leaf. The early morning weather was generally overcast, and basking was never observed. By the time the sun emerged in mid-morning, the temperature was generally warm enough (ca 26°C) that the nymphs sought out the shade.

### Sluggishness

The field survey showed that most adult individuals were no longer on the same plant the following day (N = 57). However, a few remained on the same plant for several days (N = 3): these individuals were seen on isolated individuals of infrequently used host-plant species (*Cestrum* and *S*. *acerifolium*), with no other Solanaceae nearby.

By contrast, some nymphal herds remained on the same plant for up to 3 weeks. Most groups were observed on the same plant for several days (mean: 5 +/- 1.96 S.D.), but this persistence decreased as nymphs grew (see Table 2).

The behavioural observations showed that nymphs were more active than adults, but not quite significantly so (t = 2.07; p = 0.0504; quasibinomial GLM on proportion of assay time spent moving). Nymphs spent significantly more time feeding than adults (t = 4.38; p = 0.00026; quasibinomial GLM on proportion of assay time spent feeding)–see Table 3. Both adults and nymphs spent most of the time immobile.

### Nymphal development

Nymphs exhibited a mean RGR = 0.11 +/- 0.018 S.D. mg.day^-1^.g^-1^ (growth per day divided by initial mass) over an average period of 50 days. Moults were approximately synchronized within groups: most groups moulted twice during the observation period and averaged 24 days between moults.

## Discussion

### Ontogenetic shift in defensive suite of traits

Both nymphs and adults fed exclusively on a narrow range of Solanaceae plants, consuming only mature foliage, which is generally lower in defensive compounds than are developing leaves [51]. These results do not support the hypothesis that the ontogenetic switch in defensive traits is tied to a change in diet and resulting decrease in chemical defense in the adult stage, and suggest it is more likely linked to other selection pressures associated with aggregation.

The *C*. *psittacus* nymphs remain in very conspicuous behaviourally synchronized groups, suggesting that these insects are truly gregarious sensu [30], and are not merely exhibiting resource-driven aggregation [36]. Previous research also shows that congeneric *Chromacris colorata* rejoin groups if they are experimentally separated [23]. Active gregariousness suggests that individuals derive advantages from proximity to neighbours and hence exhibit behaviours that keep them together [37]. One benefit that these nymphs likely derive from aggregation is enhancement of the aposematic signal [52]; in the context of the unusual ontogentic shift from crypsis to aposematism, we examine whether other selection pressures could also be involved in nymphal grouping. The observations provided no evidence of basking to increase body temperature, one commonly cited advantage to grouping; on the contrary, nymphs avoided direct sunlight, moving into the shade when the sun emerged. Advantages to gregariousness are thus not likely to include thermoregulation.

By contrast, nymphs did exhibit synchronized feeding, suggesting density-dependent manipulation of host quality. Synchronized feeding has been shown to increase consumption rates in several gregarious folivorous insect species [53–56]. For example, *Battus philenor* (Lepidoptera) larvae gain more weight in groups than singly, even when prevented from interacting directly, suggesting that simultaneous feeding with others improves host suitability [40], via a form of induced susceptibility involving overcoming of chemical defenses or creation of a nutrient sink [57]. Solanaceae, including *Solanum* and *Brugmansia*, contain complex mixes of alkaloids and it has been shown that, in *Brugmansia*, the tropane alkaloid scopolamine is induced following damage to leaves and inhibits subsequent herbivory [58]; synchronized feeding could limit exposure of nymphs to this noxious compound [41], providing a selective advantage to gregariousness in *C*. *psittacus* nymphs.

Nymphal development was surprisingly slow, refuting the prediction that nymphs should grow fast in order to achieve large sizes and escape invertebrate predators sooner [8,26]. Relative growth rate has not been investigated in other romaleids, but the values obtained in the present study are lower than those found for the sister family Acrididae [59–62]. Recorded development times for romaleids are shorter than those observed in the present study (see Table 1).

Both nymphs and adults *C*. *psittacus* thus exhibit adaptive suites of traits compatible with chemical defense, involving aposematism and gregariousness in the early instars, gradually shifting to crypsis and evasive flash colouration in the adult [29]. No evidence suggests that this shift is linked to lower chemical defense in adults since both feed exclusively on plants that contain compounds that can confer toxicity to vertebrate predators. A potential alternative explanation is based on bottom-up selection pressures associated with an increase in ability to metabolize host plant defenses with larger size [63]. For small nymphs, host plant defenses might favour grouping; indeed, previous work on gregarious chemically-defended insect larvae has suggested that the adaptive value of group-living in the early instars lies mainly in overcoming host plant defenses [40,64,65]. In this case, the cost of aposematism would be low since the grouped insects are already conspicuous [34,52]. In general, aposematism is favoured when toxic compounds provide effective defense and costs of crypsis are too high [1]. In the present case, feeding on toxic plants appears to provide that defense and to impose constraints that make crypsis impossible for nymphs. In adults, size-related increased ability to feed alone would imply lower host-plant-related cost for solitary living. In addition, the acquisition of flight further lowers the cost of crypsis by enabling a second line of defense based on a startle display with rapid escape [52].

### Diet breadth and defensive traits

Observations suggested no evidence of the dietary mixing seen in other romaleids: both adults and nymphs fed exclusively on a single plant species during an observation. By contrast, the gregarious aposematic *T*. *eques* and the solitary cryptic *Brachystola magna* (both Romaleidae) exhibited a high degree of individual polyphagy, with *T*. *eques* individuals feeding on up to 30 food items per day and single meals consisting of up to 11 different food items [35,66,67]. The present findings suggest that *C*. *psittacus* do not use the strategy suggested for *T*. *eques* of achieving defense against predators by mixing compounds from different host plants [67]. Conversely, being confined to a single host plant has been shown to increase efficacy of chemical defense in the polyphagous gregarious aposematic *R*. *microptera* [68]. The relative effectiveness of chemical defenses and aposematism in specialist vs generalist herbivorous insects is not clear: dietary specialization could enhance defense by increasing concentration of defensive compounds in the insect [54], or feeding from a diverse range of plants could allow synergies between plant compounds [35,69].

Solanaceae alkaloids are highly toxic to vertebrates, and are used by several aposematic Acridid grasshoppers as chemical defense [34,70,71]. Low growth rate and slow development, like those observed in the present experiment can be indicative of high chemical defenses in the host plant and associated cost of detoxification for the herbivore [62]. Indeed, even specialist feeders on toxic plants can suffer costs associated with their host plant`s defenses: for instance, alkaloids in Solanaceae host plants negatively affect development, survival and fecundity of the specialist tobacco hornworm, but do not trigger avoidance behaviour and do protect the caterpillars from predators [72]. Aposematic insects that acquire defenses from their host plant face a trade-off between top-down (predation avoidance) and bottom-up (toxicity) effects of feeding on toxic plant compounds [52,63,73]. Specialist feeding could place *C*. *psittacus* at a different point along this trade-off continuum than other diet-mixing romaleids.

The behaviour of *C*. *psittacus* nymphs and adults resembles more that of the cryptic Acridid *Schistocerca shoshone* than that recorded for aposematic romaleids: insects remain perched on the host plant rather than on the ground, move rarely and feed exclusively on that plant [74]. Chambers et al (1996) proposed alternative foraging strategies for cryptic and aposematic generalist-feeding grasshoppers, and our results suggest broadening the scope to include diet as an important driver of behavioural traits and a key bottom-up selection pressure generating correlated adaptive regimes [75].

### Defensive traits across the Romaleidae radiation

The other lubber that has been most studied, *T*. *eques*, is polyphagous and gregarious, and exhibits the common pattern of becoming more aposematic with age: young nymphs are black with faint red/yellow lines as nymphs, the yellow colouration expands as they mature leading to striking warning colouration. Adults are flightless, and have been described as very sluggish, easily captured by hand (hence the name lubber). They exude a noxious smell, suggestive of strong chemical defense. When disturbed, the adults raise their forewings to display red hindwings and make a hissing sound from the spiracles [26]. By contrast, although *Romalea microptera* and *Brachystola magna* are also polyphagous and flightless as adults, *R*. *microptera* nymphs are gregarious and aposematic and the adults exhibit broad variation in colour, whereas *B*. *magna* nymphs and adults are both solitary and cryptic [76]–see Table 1.

The romaleid toolkit of traits associated with chemical defense thus includes various different ontogenetic trajectories potentially linked to polyphagy vs oligophagy and associated effects of plant chemicals on metabolism, activity rate and development (see Table 1). Table 1 suggests a possible pattern among species with aposematic (and presumably distasteful) nymphs: some give rise to aposematic, flightless adults with noxious secretions (e.g. *T*. *eques*, *R*. *microptera*, native to the United States) whereas others (the *Tropidacris* and *Chromacris* spp of South America) mature into flighted adults with flash colouration. Little is known about the diet-breadth or distastefulness of the species with the latter strategy [28,43], but this table suggests that flight could be an important factor in adult defensive strategies. In general, romaleid defensive strategies appear very effective, as field studies record never witnessing even predation attempts [8,11,25,28].

Complex suites of traits associated with chemical defense have evolved more than once amid Orthoptera [77]. The Pyrgomorphidae (the gaudy grasshoppers) exhibit various combinations of the following traits; large size, polyphagy, flightlessness, warning colouration and nymphal gregariousness [7,78,79]. For example, *Zonocerus variegatus* nymphs are polyphagous, aposematic, gregarious and slow-growing (nymphal development typically 100–120 days) [36]. Their slow growth rate has been linked to high losses to respiration rate, suggested to be related to the ability to feed on and sequester toxic plants [80]. Feeding by *Z*. *variegatus* nymphs is facilitated by grouping and grouped nymphs grow faster than isolated individuals, particularly in the early instars, as later developmental stages show stronger tolerance of host plant compounds [80]. Similarly, *Phymateus leprosus* are aposematic, polyphagous and gregarious as nymphs. They develop very slowly (10 instars over an entire year [81]) and eventually metamorphose into very large, aposematic, polyphagous adults that are weak flyers and possess glands for defensive secretions [7,79]. Aposematism is thought to have evolved independently twice among the Pyrgomorphidae [7], and the genetic basis for feeding on toxic plants is being unraveled [79], making them an excellent parallel system to the Romaleidae for the study of defensive suites of traits.

Some Acridids are also aposematic, and some exhibit a remarkable density- and host-plant dependent aposematism, whereby nymphs that feed on chemically defended plants develop aposematic colouration at high density [71]. Similarly, desert locusts appear to switch from a solitary cryptic morph to an aposematic gregarious one according to population density [82]. This facultative aposematism has been linked to heightened costs of conspicuousness associated with aggregation on host plants that make crypsis impossible [57], in a mechanism similar to that proposed here. The Orthoptera thus include at least three families (Romaleidae, Pyrgomorphidae, Acrididae) in which aposematism appears to be linked to variable suites of traits and to follow different ontogenetic trajectories. Our results support previous work suggesting that defensive suites of traits depend not only on top-down selection pressures imposed by predators, but also on bottom-up forces from host plants, including responses to the toxicity of plant compounds.
